# Parental Knowledge, Attitudes, and Perceived Usefulness of Australian Screen Time Guidelines for Toddlers and Preschoolers

**DOI:** 10.1002/hpja.70178

**Published:** 2026-03-30

**Authors:** George Thomas, Samara Lau, Sjaan Gomersall, Michalis Stylianou, Matthew Bourke, Stephanie L. Duncombe, Ineke Vergeer, Alina Morawska, Stewart Trost, John Cairney, Stuart Biddle

**Affiliations:** ^1^ Health and Wellbeing Centre for Research Innovation, School of Human Movement and Nutrition Sciences The University of Queensland St Lucia Queensland Australia; ^2^ Australian Research Council Centre of Excellence for the Digital Child Brisbane Queensland Australia; ^3^ School of Health and Rehabilitation Sciences The University of Queensland St Lucia Queensland Australia; ^4^ School of Human Movement and Nutrition Sciences The University of Queensland St Lucia Queensland Australia; ^5^ School of Public Health The University of Queensland Herston Queensland Australia; ^6^ Centre for Health Research University of Southern Queensland Springfield Queensland Australia; ^7^ Parenting and Family Support Centre, School of Psychology The University of Queensland St Lucia Queensland Australia; ^8^ Australian Research Council Centre of Excellence for Children and Families Over the Life Course Brisbane Queensland Australia

**Keywords:** guidelines, health promotion, parenting, preschoolers, screen use, toddlers

## Abstract

**Issue Addressed:**

Managing screen use is a major concern for Australian parents of young children. However, little is known about how parents perceive, understand and appraise national screen time guidelines for children aged between 1 and 5 years. This study examined parental knowledge, attitudes and perceived usefulness of the Australian screen time guidelines and explored associations with sociodemographic factors.

**Methods:**

Data were drawn from a cross‐sectional online survey of Australian parents (*n* = 229) of young children aged between 1 and 5 years. Knowledge of screen time guidelines (multiple‐choice item), attitudes (4 items; 5‐point scale) and perceived usefulness (3 items; 5‐point scale) were examined. Descriptive statistics were calculated for independent variables and regression models estimated to examine sociodemographic factors associated with knowledge, attitudes and perceived usefulness.

**Results:**

Parents of toddlers were 4.45 times (*p* < 0.001) more likely to know the screen time guidelines than parents of preschoolers. Knowledge was lower among parents in full‐time employment and those living in major cities (*p* < 0.05). Attitudes did not differ by demographics, but parents of girls rated the guidelines as more useful than parents of boys (*p* = 0.038). Higher attitude scores (*p* = 0.017) and greater perceived usefulness (*p* = 0.047) were associated with correctly identifying the guidelines.

**Conclusion:**

Knowledge gaps exist, particularly among parents of preschoolers, those employed full‐time and those living in major cities. Targeted education with diverse parent groups is needed to ensure guidelines are both understood and perceived as useful.

**So What?:**

Inclusive guidelines, supported by tailored education and implementation strategies for key parent groups, may improve understanding and uptake in realistic family contexts.

## 1 Introduction

In today's world, screens – smartphones, televisions, tablets and computers – are deeply embedded in daily family life [1]. Device ownership begins early, with 40% of children having access to their own tablet by age two, increasing to 57% by age four [2]. In Australia, screen use in early childhood is substantial, with toddlers under 2 years averaging 14 h per week viewing screens, while children aged 2–5 years spending on average 26 h per week on screen‐based devices [3]. At the same time, the nature of screen engagement is changing. Traditional television viewing is declining, with a shift towards interactive formats such as educational platforms and short‐form video apps [2]. Once used primarily for passive entertainment or learning, screens are now woven into daily routines, helping with transitions like bedtime or providing downtime [4].

This rapid evolution of screen use has raised concerns among parents regarding its potential impact on children's development, health and wellbeing [5]. In 2021, more than 90% of Australian parents identified excessive screen time as a top health concern [6], underscoring the urgency of addressing this issue. Historically, prolonged screen use has been associated with negative outcomes for learning, physical health and social development [7, 8]. However, recent evidence suggests a more complex association between screen use and health and education outcomes. As highlighted in a large‐scale analysis of harmonised data from over 100 meta‐analyses, the negative impacts of general screen use and social media (e.g., learning, literacy, body composition and depression) were moderated by content type (e.g., educational programming had better outcomes than general screen use) and context (e.g., co‐viewing with a parent had better outcomes than general screen use) [9]. Such findings highlight the need for greater guidance to help parents navigate this complex digital landscape and optimise their children's engagement with screens.

Recommendations for young children's screen use were first introduced in the *Australian Physical Activity Guidelines for Children aged 0–5 years*, released by the Australian Government in 2010 [10]. These guidelines advised no screen exposure for children under 2 years and limiting screen use to less than one hour per day for those aged 2–5 years [10]. Evidence at the time suggested that many parents found these limits impractical and unrealistic [11]. In response, these recommendations were later incorporated into the *24‐Hour Movement Guidelines for the Early Years*, which frame screen use alongside physical activity, sedentary behaviour and sleep [12]. Although the specific screen‐time limits remain unchanged, this update reflected a broader recognition that daily movement behaviours are interdependent and collectively influence children's health and development [13]. Nonetheless, debate continues, with some arguing that screen‐time guidance should be separated from movement recommendations, underscoring the complexity of this evolving field [14].

As primary regulators of young children's screen use, parents play a critical role in interpreting and implementing screen time guidelines. Parental knowledge is particularly influential, as it underpins the strategies they use to manage children's screen behaviours, such as setting rules, supervising use and promoting alternative activities like physical play and reading [15, 16]. Evidence shows that toddlers whose parents accurately understood the 24‐h movement guidelines were over eight times more likely to meet the screen time recommendation compared to toddlers whose parents had no knowledge of guidelines [17]. In addition, parents' perceptions of how practical screen time guidelines are appear crucial for how they manage screen use. When recommendations are seen as unrealistic or unclear, parents are less likely to follow them, often turning instead to alternative sources such as parenting forums or online advice [18]. Importantly, research also suggests that young children's (0–5 years) adherence to guidelines may vary according to parental sociodemographic factors, such as education level, maternal age and socioeconomic status [19]. These findings highlight the need for more nuanced research into how different groups of parents understand and apply screen time guidelines.

Despite the evolution of screen time guidelines in recent years, little is known about Australian parents' awareness of, attitudes towards and perceived usefulness of current national screen time guidelines for children under 5 years. To date, the majority of research in this area has been conducted outside of Australia (e.g., Canada) [20] or has referenced outdated guidelines [11]. Research in Australia has primarily focused on parents of infants (< 2 years) [17] or on parents of primary school‐aged children (5–12 years) [21], with less attention given to preschool‐aged children (3–5 years). The toddler and preschool years represent a critical developmental period during which early experiences, including screen exposure, can have lasting impacts on cognitive, social and emotional trajectories [22]. Therefore, it is important to examine how Australian parents of young children engage with current screen time guidelines to ensure they are both relevant and practical.

While parental knowledge of screen time recommendations is often emphasised [23], fewer studies have explored parents' attitudes towards the guidelines or their perceived usefulness, particularly in relation to specific elements such as wording, content and practical relevance [24]. These gaps limit our understanding of how national recommendations are received and interpreted by families, potentially reducing the guidelines' real‐world applicability and effectiveness. There is a pressing need for research that specifically examines how parents of toddlers and preschoolers understand and engage with the current screen time guidelines, to ensure that recommendations are appropriately communicated and effectively implemented during this formative stage. Accordingly, this study examined Australian parents' awareness, attitudes and perceived usefulness of the national screen time guidelines for children aged between 1 and 5 years and explored whether these perceptions differed according to key sociodemographic characteristics.

## 2 Methods

### 2.1 Recruitment

Eligible participants were parents/carers (18 years or older) living in Australia with children between 1 and 5 years of age. Hereafter, we use the term ‘parents’ to refer inclusively to both parents and carers (inclusive of all primary caregivers, including guardians and other family members involved in raising the child). Participants were recruited through crowdsourcing techniques, social media platforms, flyers and word of mouth. Parenting forums and websites, professional networks and local childcare centres were also contacted to facilitate recruitment. Recruitment materials included a QR code which directed interested participants to an online information sheet, consent form and link to the online survey. Participants provided informed consent prior to completing the survey. At the end of the survey, participants could enter a draw to win a gift voucher. Participants who were interested to enter the prize draw could ‘opt in’ by providing their email address, which was recorded separately from their survey responses to maintain anonymity. Ethical approval was granted by The University of Queensland's Human Research Ethics Committee (HE001805).

### 2.2 Data Collection

Participants completed an online survey, administered via Qualtrics, between May and September 2024. This study drew from a broader online survey examining challenges and supports related to children's screen use. The survey took a median time of 15 min and 40 s to complete. The current paper focuses on measures of parental knowledge, attitudes and perceived usefulness towards national screen time guidelines, along with household, parental and child sociodemographic characteristics. Where parents had multiple children under the age of 5 years, they were directed to respond to the survey for the youngest child aged between 1 and 5 years. For example, if a parent had children aged 2, 4 and 7 years, they were asked to respond regarding their 2‐year‐old. Parents with an oldest child of < 1 year of age were excluded, as screen use habits during infancy are likely to differ substantially from those of toddlers (1–2 years) and preschool‐aged children (3–5 years). Only parents with complete data on study variables (i.e., knowledge, attitudes, perceived usefulness and demographics) were included in the current study.

### 2.3 Measures

Survey items were developed to assess parental knowledge, attitudes and perceived usefulness of the Australian screen time guidelines. Item content was informed by the structure and wording of the current national guidelines. Inclusion was guided by alignment with the study aims and feasibility considerations to minimise participant burden. Items were refined through interviews with parents and an extensive expert panel review that included individuals with expertise in child health, screen use research and survey design. Items were piloted with a small group of parents (*n* = 10), which led to refinements in wording, response options and item order. This iterative process aligns with best practice in survey development and validation, ensuring content validity and parent‐centred applicability [25].

#### 2.3.1 Sociodemographic and Household Characteristics

Parents reported the child's age and gender, as well as information about themselves, including relationship to the child (mother; father; other), age (≤ 30 years; 31–35 years; 36–40 years; > 40 years), higher education level (yes; no), employment status (employed full‐time; employed part‐time; unemployed; other), country of birth (Australia; other), language spoken at home (only English; other) and Indigenous status (Indigenous; non‐Indigenous). Parents were also asked to report on their household composition, including the number of parents/carers (one; two; three or more) and sibling status (only child; siblings). For the purposes of this paper, siblings refer to the children under 18 years living within the home regardless of if they are siblings by birth, stepsiblings, foster siblings, or other children within the home. Participants were also asked to provide their postcode of residence and from these data, area‐level socioeconomic status was calculated based on the Index of Relative Socioeconomic Advantage and Disadvantage (IRSAD) [26]. For this study, IRSAD deciles were clustered into three groups: high disadvantage (deciles 1–3), mid‐range (deciles 4–7) and low disadvantage (deciles 8–10). Additionally, participant postcodes were used to determine the regionality of participants based on the Modified Monash Model and were categorised as major cities (MM1), regional centres (MM2), rural town (MM3‐5) and remote/very remote (MM6‐7).

#### 2.3.2 Knowledge of Australian Screen Time Guidelines for Toddlers and Preschoolers

Parental knowledge of Australian screen time guidelines for toddlers (1–2 years) and preschoolers (3–5 years) was assessed using the question: ‘*What are the current Australian screen time guidelines for your youngest child aged 1 year or above*?’ Respondents were asked to select one answer from a multiple‐choice list, with responses subsequently dichotomised into ‘Correct’ (correct selection of the recommended guidelines) or ‘Incorrect’ (any other response). Parents were only shown guideline options relevant to the age of their youngest child (i.e., toddlers aged 2 years or younger, or preschoolers aged 3 years or older). A complete list of response options and participant answers related to guideline knowledge is provided in Table S1. After providing their response, parents were then shown the correct guideline.

#### 2.3.3 Parent Attitudes Towards Australian Screen Time Guidelines for Toddlers and Preschoolers

Parent attitudes towards the guidelines were assessed by measuring the extent to which parents agreed with four statements on a 5‐point Likert scale (1 = strongly disagree to 5 = strongly agree). The statements evaluated four aspects, including liking (e.g., ‘I like the current screen time guidelines’), satisfaction (e.g., ‘Overall, I am satisfied with the current screen time guidelines’), suitability (e.g., ‘The current screen time guidelines seem suitable for me’) and ease of understanding (e.g., ‘I find the current screen time guidelines easy to understand’). Scores were summed to create a total score for analysis. The total score showed excellent internal consistency (*α* = 0.93) in the current sample.

#### 2.3.4 Perceived Usefulness of Australian Screen Time Guidelines for Toddlers and Preschoolers

Perceived usefulness of the guidelines was assessed using three statements on a 5‐point adjective scale (1 = not at all useful to 5 = very useful). The statements evaluated three separate aspects, including recommended screen time limits (e.g., ‘The amount of screen time allowed [e.g., hours per day]’), the wording (e.g., ‘The wording and content of the current screen use guidelines’) and guidance provided about screen use (e.g., ‘Information about how/with whom/when screens should be used’). Scores were summed to create a total score for analysis. The total score showed excellent internal consistency (*α* = 0.91) in the current sample.

### 2.4 Data Analysis

Data were analysed using IBM SPSS Statistics (Version 29.0.1.0; IBM Corp., Armonk, NY, USA). Missing data were examined, and the distributions of continuous variables were assessed for skewness and kurtosis. As no substantial deviations were observed, normality was assumed. Descriptive statistics were calculated for all variables, including frequencies and proportions for categorical variables and means and standard deviations for continuous variables. Binary logistic regression models were estimated to examine differences in knowledge of the toddler and preschooler screen time guidelines and determine if knowledge differs across demographic characteristics. Independent samples *t*‐tests and one‐way ANOVA were conducted to examine differences in parental attitudes towards the guidelines and perceived usefulness across demographic groups. Where omnibus ANOVA effects were statistically significant, pre‐specified pairwise comparisons were performed using the protected Fisher's Least Significant Difference (LSD) procedure. Effect sizes were calculated using Cohen's d, with values of 0.2, 0.5 and 0.8 interpreted as small, medium and large effects, respectively [27]. The association between perceived attitudes towards guidelines and perceived usefulness of guidelines with guideline knowledge were estimated using logistic regression. To improve interpretability, perceived attitudes and usefulness were *z*‐score standardised to have a mean of zero and a standard deviation of one. These models controlled for which guideline parents were asked about (toddler vs. preschooler), and any variables that were independently related to either guideline knowledge, perceived attitudes, or perceived usefulness. All statistical tests were two‐tailed with significance set at *p* < 0.05.

## 3 Results

Of the 480 parents who consented to participate, 102 responses were flagged by Qualtrics' reCAPTCHA system (score ≤ 0.5), indicating a higher likelihood of automated (bot) entries or low‐quality responses (e.g., rapid completion, straight‐lining, or nonsensical answers). Manual inspection of the remaining responses identified an additional 18 invalid cases. These responses were excluded, leaving 360 valid entries. Of these, 64 participants did not progress beyond the initial demographics section and were subsequently removed from analyses. Of 296 parents who completed the survey, 53 responses were excluded due to missing data on key outcome variables (knowledge, attitudes, or perceived usefulness of screen time guidelines). An additional 14 participants were excluded due to missing demographic data. In the current study, the final analytical sample included 229 participants.

### 3.1 Sample Characteristics

Table 1 provides a summary of participant sociodemographic characteristics. The sample included 122 parents of toddlers (1–2 years) and 107 parents of preschool‐aged children (3–5 years). Just over half of the participants' youngest child aged 1–5 years were girls (52.0%), with a mean age of 2.6 years (SD = 1.2). Most participants were mothers (90.8%), non‐Indigenous (85.2%), in full‐time (41.9%) or part‐time employment (41.5%), university‐educated (83.8%), and born in Australia (81.2%), with English being their primary language (88.2%). Participant ages ranged between 23 and 50 years (M = 37.6; SD = 4.8). Most participant households had two parents/carers (91.7%) and had more than one child (57.6%). Participants came from diverse socioeconomic areas, with 44.1%, 40.6% and 15.3% from areas of low‐, mid‐ and high‐ relative disadvantage, respectively.

**TABLE 1:** Sample characteristics among 229 parents of young children aged 1–5 years.

Characteristics	*n* (%)
Child demographics	
Gender	
Girl	119 (52.0)
Boy	110 (48.0)
Age (years)	
1	42 (18.3)
2	80 (34.9)
3	51 (22.3)
4	37 (16.2)
5	19 (8.3)
Parent demographics	
Relationship to child	
Mother	208 (90.8)
Father	21 (9.2)
Age (years)	
≤ 30	19 (8.3)
31–35	54 (23.6)
36–40	93 (40.6)
> 40	63 (27.5)
Indigenous status	
Non‐indigenous	195 (85.2)
Indigenous	34 (14.8)
Employment status	
Full‐time employed	96 (41.9)
Part‐time employed	95 (41.5)
Unemployed	15 (6.6)
Other	23 (10.0)
Higher education	
Yes	192 (83.8)
No	37 (16.2)
Country of birth	
Australia	186 (81.2)
Other	43 (18.8)
Language	
English	202 (88.2)
Other	27 (11.8)
Household characteristics	
Number of parents/carers	
1	14 (6.1)
2	210 (91.7)
3+	5 (2.2)
Number of siblings	
Only‐child	97 (42.4)
Siblings	132 (57.6)
Area‐level SES	
Low disadvantage	101 (44.1)
Mid disadvantage	93 (40.6)
High disadvantage	35 (15.3)
Region	
Major cities	193 (84.3)
Regional centres	24 (10.5)
Rural towns	10 (4.4)
Remote/very remote	2 (0.9)

Abbreviation: SES = socioeconomic status.

### 3.2 Knowledge of Screen Time Guidelines

Overall, 74.2% of parents correctly identified the guideline relevant to their child's age. Parents of toddlers had 4.45 times higher odds (95% CI = 2.32, 8.55, *p* < 0.001) of knowing the screen time guidelines (89.6% correct) compared to parents of preschoolers (59.8%). Table 2 provides the proportion of parents who correctly identified the screen time guideline relevant to their child's age group. Parents aged 36–40 years (*p* < 0.05), those in part‐time employment (*p* < 0.05), and those living in regional, rural or remote communities (*p* < 0.001) were more likely to know the guidelines, while Indigenous parents were less likely to know the guidelines than non‐Indigenous parents (*p* < 0.05). After adjusting for guideline category (toddler or preschooler), employment status and region remained significant, with parents employed part‐time (AOR = 2.19, 95% CI: 1.09–4.39, *p* = 0.027) and parents living in regional, rural or remote communities (AOR = 3.20, 95% CI: 1.05–9.76, *p* = 0.042) more likely to correctly identify the guidelines than those in full‐time employment and living in major cities. No other sociodemographic differences were observed.

**TABLE 2:** Parental knowledge of toddler and preschooler screen time guidelines by demographic characteristics.

	% correct	OR (95% CI)	AOR (95% CI)
Child demographics			
Gender			
Girl	74.5%	Ref	Ref
Boy	73.9%	0.97 (0.54, 1.75)	0.94 (0.52, 1.82)
Parent demographics			
Relationship to child			
Mother	76.0%	Ref	Ref
Father	57.1%	0.42 (0.17, 1.06)	0.42 (0.16, 1.12)
Age (years)			
< 30	78.9%	2.01 (0.60, 6.81)	1.34 (0.37, 4.88)
31–35	74.1%	1.53 (0.69, 3.41)	1.06 (0.45, 2.49)
36–40	76.9%	**2.09 (1.01, 4.31)**	1.78 (0.83, 3.80)
> 40	65.1%	Ref	Ref
Indigenous status			
Non‐indigenous	76.9%	Ref	Ref
Indigenous	58.8%	**0.43 (0.20, 0.92)**	0.48 (0.22, 1.08)
Employment status			
Full‐time	65.6%	Ref	Ref
Part‐time	81.1%	**2.24 (1.15, 4.35)**	**2.19 (1.09, 4.39)**
Unemployed	80.0%	2.10 (0.55, 7.95)	1.87 (0.47, 7.57)
Other	78.3%	1.89 (0.64, 5.53)	1.53 (0.49, 4.76)
Higher education			
Yes	81.1%	Ref	Ref
No	72.9%	0.63 (0.26, 1.52)	0.64 (0.25, 1.61)
Country of birth			
Australia	75.8%	Ref	Ref
Other	67.8%	0.66 (0.32, 1.36)	0.68 (0.32, 1.47)
Language			
English	75.2%	Ref	Ref
Other	66.7%	0.66 (0.28, 1.56)	0.64 (0.26, 1.60)
Household characteristics			
Number of parents			
Dual parent	74.3%	Ref	Ref
Other	73.7%	0.97 (0.33, 2.82)	0.89 (0.29, 2.75)
Siblings			
Yes	75.0%	Ref	Ref
No	73.2%	0.91 (0.50, 1.66)	0.92 (0.49, 1.72)
Area level SES			
Low disadvantage	73.3%	Ref	Ref
Mid disadvantage	71.0%	0.89 (0.15, 1.67)	0.94 (0.48, 1.81)
High disadvantage	85.7%	2.19 (0.77, 6.22)	2.02 (0.69, 5.97)
Region			
Major city	71.5%	Ref	Ref
Regional/rural/remote	88.9%	**3.19 (1.08, 9.44)**	**3.20 (1.05, 9.76)**

*Note:* Adjusted for guidelines age category (i.e., toddler or preschooler). Bolded values indicate statistically significant differences (*p* < 0.05).

Abbreviations: AOR = adjusted odds ratio; OR = odds ratio; SES = social economic status.

### 3.3 Attitudes Towards and Perceived Usefulness of Screen Time Guidelines

Most parents reported positive attitudes towards the Australian screen time guidelines for toddlers and preschoolers (Table 3). Approximately two‐thirds agreed or strongly agreed that they liked the guidelines (63.3%), were satisfied with them (68.2%), found them suitable (64.6%), and considered them easy to understand (71.6%), while around one‐quarter gave neutral responses across items (21.0%–28.8%). Perceived usefulness followed a similar pattern (Table 4). Around two‐thirds of parents rated the recommended amount of screen time (69.0%) and the wording/content of the guidelines (60.7%) as useful or very useful, while just over half (56.3%) considered information about how, with whom and when screens should be used as useful. Neutral responses were common, ranging from 22.7% to 31.4% across items.

**TABLE 3:** Attitude responses in relation to Australian screen time guidelines for toddlers and preschoolers.

Statement	Strongly disagree (1)	Disagree (2)	Neutral (3)	Agree (4)	Strongly agree (5)	M (SD)
I like the current screen uses guidelines	4 (1.7)	14 (6.1)	66 (28.8)	95 (41.5)	20 (21.8)	3.76 (0.92)
Overall, I am satisfied with the current screen use guidelines	3 (1.3)	17 (7.4)	53 (23.1)	111 (48.5)	45 (19.7)	3.78 (0.89)
The current screen use guidelines seem suitable for me	6 (2.6)	27 (11.8)	48 (21.0)	104 (45.4)	44 (19.2)	3.67 (1.00)
I find the current screen use guidelines easy to understand	1 (0.4)	5 (2.2)	59 (25.8)	113 (49.3)	51 (22.3)	3.91 (0.78)
Total attitudes (possible range: 4–20)						15.11 (3.05)

*Note:* Results presented as *n* (%) unless otherwise stated.

Abbreviation: SD = standard deviation.

**TABLE 4:** Perceived usefulness responses in relation to Australian screen time guidelines for toddlers and preschoolers.

Statement	Not at all useful (1)	Not useful (2)	Neutral (3)	Agree (4) useful	Very useful (5)	M (SD)
The amount of screen time allowed (e.g., hours per day)	5 (2.2)	14 (6.1)	52 (22.7)	112 (48.9)	46 (20.1)	3.79 (0.91)
The wording and content of the current screen use guidelines	3 (1.3)	15 (6.6)	72 (31.4)	113 (49.3)	26 (11.4)	3.63 (0.82)
Information about how/with whom/when screens should be used	8 (3.5)	21 (9.2)	71 (31.0)	102 (44.5)	27 (11.8)	3.52 (0.94)
Total attitudes (possible range: 3–15)						10.93 (2.16)

*Note:* Results presented as *n* (%) unless otherwise stated.

Abbreviation: SD = standard deviation.

No significant differences in attitude scores were observed by living arrangements, parental education, child's gender, parent relation to child, Indigenous status, language, country of birth, employment status, or regionality (Table 5). Parents of girls rated the guidelines as more useful than parents of boys (MD = 0.59, *t*(227) = 2.09, *p* = 0.038, *d* = 0.28). Perceived usefulness varied by parent age (F (3) = 3.19, *p* = 0.025), with parents aged 36–40 years rating guidelines as more useful than those aged 31–35 (MD = 0.95, *p* = 0.009, *d* = 0.47) and those over 40 years (MD = 0.87, *p* = 0.009, *d* = 0.42). No other demographic differences in perceived usefulness were identified.

**TABLE 5:** Attitudes towards and perceived usefulness of guidelines by demographic characteristics.

	Attitude		Perceived usefulness	
	M (95% CI)	*p*	M (95% CI)	*p*
Child demographics				
Gender		0.209		**0** **.** **038**
Girl	15.35 (14.81, 15.90)		11.22 (10.85, 11.59)	
Boy	14.85 (14.26, 15.43)		10.63 (10.20, 11.05)	
Age (years)		0.908		0.855
Toddler (1, 2)	15.13 (14.59, 15.67)		10.96 (10.54, 11.38)	
Preschool (3–5)	15.08 (14.49, 15.68)		10.91 (10.54, 11.27)	
Parent demographics				
Relationship to child		0.585		0.721
Mother	15.14 (14.72, 15.57)		10.92 (10.62, 11.22)	
Father	14.76 (13.68, 15.85)		11.10 (10.30, 11.90)	
Age (years)		0.380		**0.** **025**
< 30	15.26 (14.13, 16.40)		10.95 (9.79, 12.10)	
31–35	14.65 (13.86, 15.43)		10.48 (9.89, 11.07)	
36–40	15.50 (14.87, 16.11)		11.44 (11.04, 11.84)	
> 40	14.89 (14.03, 15.75)		10.57 (10.00, 11.14)	
Indigenous status		0.405		0.653
Non‐Indigenous	15.18 (14.73, 15.63)		10.91 (10.59, 11.22)	
Indigenous	14.71 (13.98, 15.43)		10.93 (10.65, 11.22)	
Employment status		0.278		0.333
Full‐time	14.67 (14.16, 15.17)		10.75 (10.33, 11.17)	
Part‐time	15.40 (14.65, 16.15)		10.98 (10.50, 11.46)	
Unemployed	15.13 (13.13, 16.83)		10.73 (9.97, 11.50)	
Other	15.74 (14.76, 16.72)		11.65 (10.76, 12.54)	
Higher education		0.195		0.060
Yes	15.22 (14.78, 15.66)		11.05 (10.75, 11.35)	
No	14.51 (13.58, 15.45)		10.32 (9.56, 11.09)	
Country of birth		0.838		0.490
Australia	15.13 (14.68, 15.58)		10.89 (10.56, 11.21)	
Other	15.02 (14.15, 15.89)		11.14 (10.62, 11.66)	
Language		0.204		0.833
English	15.20 (14.78, 15.62)		10.95 (10.64, 11.25)	
Other	14.41 (13.12, 15.70)		10.85 (10.19, 11.52)	
Household characteristics				
Number of parents		0.392		0.361
Dual parent	15.06 (14.64, 15.47)		10.90 (10.60, 11.19)	
Other	15.68 (14.30, 17.07)		11.37 (10.31, 12.42)	
Siblings		0.368		0.434
Yes	15.27 (14.73, 15.80)		11.03 (10.66, 11.40)	
No	14.90 (14.29, 15.50)		10.80 (10.37, 11.24)	
Area level SES		0.279		0.935
Low disadvantage	15.43 (14.85, 16.00)		10.98 (10.57, 11.39)	
Mid disadvantage	14.99 (14.34, 15.64)		10.87 (10.42, 11.31)	
High disadvantage	14.51 (13.46, 15.57)		10.97 (10.14, 11.81)	
Region		0.862		0.299
Major city	15.12 (14.69, 15.56)		10.87 (10.58, 11.16)	
Regional/rural/remote	15.03 (14.00, 16.05)		11.28 (10.38, 12.17)	

*Note:* Bolded values indicate statistically significant differences (*p* < 0.05).

Abbreviations: CI = confidence interval; M = mean; SES = social economic status.

### 3.4 Association Between Screen Time Guidelines Knowledge and Parent Attitudes and Perceived Usefulness

There was a significant positive association between perceived attitudes towards guidelines and guideline knowledge. Parents with a one standard deviation higher score for perceived attitudes towards the guidelines were 1.5 times more likely to correctly identify the screen time guidelines (AOR = 1.49, 95% CI = 1.07, 2.06, *p* = 0.017). There was also a significant positive relationship between perceived usefulness of the guidelines and guideline knowledge. Parents with a one standard deviation higher score for perceived usefulness of the guidelines were 1.4 times more likely to correctly identify the screen time guidelines (AOR = 1.40, 95% CI = 1.01, 1.95, *p* = 0.047).

## 4 Discussion

The study explored Australian parents' knowledge, attitudes and perceived usefulness of national screen time guidelines for children aged 1–5 years and examined associations with sociodemographic characteristics. Overall, 74.2% of parents correctly identified the guideline relevant to their child's age. Parents of toddlers had over 4 times higher odds of knowing the screen time guidelines than parents of preschoolers. Further analysis revealed greater knowledge among parents in part‐time employment and those living in regional, rural, or remote regions. Most parents reported favourable attitudes towards the guidelines as well as general positive perceptions of their usefulness. Although attitudes did not vary significantly by demographic factors, parents of girls rated the guidelines as more useful than parents of boys.

The higher proportion of parents of toddlers correctly identifying the screen time guideline aligns with previous research in Canada [20]. Parents of toddlers may be more proactive in seeking information about screen use as they navigate early parenting concerns and routines [28]. The transition into parenthood often prompts heightened information‐seeking behaviours, particularly around developmental milestones and health behaviours like screen exposure [29]. Additionally, this may reflect the increased frequency of health checks scheduled in the lead up to toddler years, compared to the preschooler years [30]. These checks represent critical touchpoints for delivering parenting guidance, including raising awareness of screen time recommendations; reduced availability of such checks during the child's preschooler years may limit exposure to this information. Promising policy developments – such as expanded child health checks in early childhood education settings [31] – offer infrastructure and timely opportunities to deliver screen time guidance to parents of preschoolers. However, this will require equipping health professionals with clear, evidence‐based messaging that parents find practical and educational [32].

Social determinants appeared to be important in shaping parental knowledge. In the adjusted analysis, parental employment status and region of residence were associated with knowledge of the guidelines. Parents employed part‐time had greater odds of correctly identifying the guidelines compared with those in full‐time employment. This finding differs from evidence reported in Canada, where full‐time working parents were actually more knowledgeable of the guidelines [20]. Longer work hours and limited flexibility commonly associated with full‐time employment may contribute to time constraints, thereby reducing opportunities to access or engage with health messaging. Parents living in regional, rural or remote communities also had greater odds of correctly identifying the guidelines than those living in major cities. Evidence examining geographic differences in parental awareness of screen time guidelines is limited, and further research is needed to better understand how geographic context may influence parents' awareness of public health guidance.

Parents of girls perceived the guidelines as more useful than parents of boys, potentially reflecting gendered social norms. Prior research suggests that parents may subconsciously apply different expectations to boys and girls, influencing their receptivity to screen time recommendations [33]. This perceived usefulness may affect uptake, with parents of girls potentially more inclined to align with guideline recommendations, as supported by Australian data showing that 28% of girls aged 2–5 years met screen time guidelines compared to 22% of boys [19]. These findings highlight the subtle ways in which gender norms may influence the perceived applicability (and subsequent implementation) of public health recommendations.

Interestingly, parents rated information on how, with whom and when screens should be used as the least useful aspect of the guidelines. This is notable given that contextual factors such as co‐use, content quality and timing, are increasingly recognised as important factors of screen use outcomes [9]. However, evidence in the literature remains mixed. For example, while some studies associate co‐viewing with enhanced language development [34], others have linked television co‐viewing with lower adherence to screen time limits [17]. Such inconsistencies may contribute to parental uncertainty about how to translate guidelines into everyday practice. Providing concrete, accessible examples of screen use contexts (e.g., high‐quality co‐viewing versus background TV, or limits on evening use) may enhance perceived relevance. It should be noted that around one‐third of parents gave a neutral response when asked about the usefulness of guidelines, suggesting some ambivalence about the guideline's utility. Guideline development must carefully balance nuance with clarity to support parents in applying recommendations confidently and consistently [14].

Parents who correctly identified the guidelines reported significantly more positive attitudes and greater perceived usefulness, congruent with previous findings among parents of younger children [17]. This may reflect greater parent involvement in managing screen use, particularly as children gain autonomy and routines are being established [15]. This presents an opportunity for interventions that leverage these existing motivations. Parents who are aware of the guidelines may be more motivated to seek and apply health recommendations, or conversely, perceiving the guidelines as relevant and practical may enhance recall and uptake [35]. These findings suggest that clarity and perceived credibility of the guidelines are important determinants of parental engagement.

A key strength of this study is its inclusion of parents of both toddlers and preschoolers, offering a broad view across early childhood. Beyond documenting parental knowledge, we also examined attitudes, perceived usefulness and their associations with social demographics to provide new insights into how these factors interact and influence parental engagement with screen time guidelines. To our knowledge, this is one of the first Australian studies to link guideline knowledge with both perceived attitudes and usefulness, extending the international evidence base and underscoring the importance of education and implementation strategies for improving uptake of guidelines [20]. However, limitations must be acknowledged. First, the sample, while diverse, excluded parents of children under 1 year and was skewed towards highly educated mothers, which may limit generalisability. For example, the relatively high levels of guideline knowledge observed may partly reflect the educational profile of the sample. Future research should prioritise recruiting more socioeconomically and educationally diverse samples, including parents of children under 1 year. Second, the size of categories for some demographic variables was imbalanced, and there were several instances of small sample sizes within categories. Therefore, the power of many of the statistical comparisons may have been low, limiting our ability to detect small‐to‐moderate, albeit potentially meaningful, differences in outcomes between categories. Third, the approach to participation, which relied exclusively on a QR code and an online survey, may have limited uptake among individuals who were less familiar or comfortable with these methods. Finally, household income and detailed employment characteristics, which may also impact screen time, were not collected. Future research should prioritise more representative sampling and including these demographic characteristics to ensure findings reflect the full spectrum of Australian families.

## 5 Conclusion

This study found disparities in parents' knowledge and engagement with screen time guidelines, with higher knowledge among parents of toddlers, those in part‐time employment, and those living in regional, rural, or remote regions. Greater knowledge was linked to more positive attitudes and perceived usefulness. Findings highlight the need for equity‐focused education and tailored implementation strategies, particularly for key population groups. While guidelines already vary by age, supportive resources that address contextual factors (e.g., co‐use, content quality, sibling age differences) and are co‐designed with parents may enhance clarity, relevance and uptake. Such an approach shifts the focus from maximum time limits towards the quality of screen engagement and effective parenting practices that fit within realistic family contexts.

## Funding

G.T. and S.G. are supported by the Health and Wellbeing Centre for Research Innovation (HWCRI), co‐funded by The University of Queensland (UQ) and the Queensland Government through Health and Wellbeing Queensland. This project was supported by a UQ HMNS Small Research Grant.

## Ethics Statement

Ethics approval was obtained from The University of Queensland's Low Risk Human Research Ethics Committee (2023/HE001805).

## Conflicts of Interest

The Parenting and Family Support Centre is partly funded by royalties stemming from published resources of the Triple P – Positive Parenting Program, which is developed and owned by The University of Queensland (UQ). Royalties are also distributed to the Faculty of Health and Behavioural Sciences at UQ and contributory authors of published Triple P resources. Triple P International (TPI) Pty Ltd. is a private company licensed by UQ to publish and disseminate Triple P worldwide. The authors of this report have no share or ownership of TPI. Prof Morawska receives royalties from TPI. TPI had no involvement in the study design, collection, analysis or interpretation of data, or writing of this report. Prof Morawska is an employee at UQ.

## Supporting information


**Table S1:** presents survey items and responses assessing parental knowledge of Australian screen time guidelines.

## Data Availability

The data that support the findings of this study are available from the corresponding author upon reasonable request.

## References

[1] R. Barr , “Growing Up in the Digital Age: Early Learning and Family Media Ecology,” Current Directions in Psychological Science 28, no. 4 (2019): 341–346. 31423053 10.1177/0963721419838245 PMC6697422

[2] S. Mann , A. Calvin , A. Lenhart , and M. B. Robb , The Common Sense Census: Media Use by Kids Zero to Eight, 2025 (Common Sense Media, 2025).

[3] A. Rhodes , Screen Time: What's Happening in Our Homes? Australian Child Health Poll (Royal Children's Hospital, 2017).

[4] A. G. LeBlanc , K. E. Gunnell , S. A. Prince , T. J. Saunders , J. D. Barnes , and J. P. Chaput , “The Ubiquity of the Screen: An Overview of the Risks and Benefits of Screen Time in Our Modern World,” Translational Journal of the American College of Sports Medicine 2, no. 17 (2017): 104–113.

[5] A. H. Joshi and T. Hinkley , Too Much Time on Screens? Screen Time Effects and Guidelines for Children and Young People (Australian Institute of Family Studies, 2021).

[6] A. Rhodes , Top 10 Child Health Problems: What Australian Parents Think. Australian Child Health Poll (Royal Children's Hospital, 2021).

[7] V. Carson and I. Janssen , “Associations Between Factors Within the Home Setting and Screen Time Among Children Aged 0‐5 Years: A Cross‐Sectional Study,” BMC Public Health 12 (2012): 539. 22823887 10.1186/1471-2458-12-539 PMC3439297

[8] N. Stiglic and R. M. Viner , “Effects of Screentime on the Health and Well‐Being of Children and Adolescents: A Systematic Review of Reviews,” BMJ Open 9, no. 1 (2019): e023191. 10.1136/bmjopen-2018-023191 PMC6326346 30606703

[9] T. Sanders , M. Noetel , P. Parker , et al., “An Umbrella Review of the Benefits and Risks Associated With Youths' Interactions With Electronic Screens,” Nature Human Behaviour 8, no. 1 (2024): 82–99. 10.1038/s41562-023-01712-8 37957284

[10] Australian Government Department of Health and Aging , National Physical Activity Recommendations for Children 0–5 Years (Commonwealth of Australia, 2010).

[11] A. Brown and E. Smolenaers , “Parents' Interpretations of Screen Time Recommendations for Children Younger Than 2 Years,” Journal of Family Issues 39, no. 2 (2016): 406–429.

[12] Australian Government Department of Health and Aged Care , Physical Activity and Exercise Guidelines for All Australians: For Infants, Toddlers and Preschoolers (Birth to 5 Years) (Commonwealth of Australia, 2021).

[13] A. D. Okely , D. Ghersi , K. D. Hesketh , et al., “A Collaborative Approach to Adopting/Adapting Guidelines–The Australian 24‐Hour Movement Guidelines for the Early Years (Birth to 5 Years): An Integration of Physical Activity, Sedentary Behavior, and Sleep,” BMC Public Health 17, no. 5 (2017): 869. 29219094 10.1186/s12889-017-4867-6 PMC5773882

[14] L. Straker , S. Edwards , L. Kervin , J. Burley , D. Hendry , and D. Cliff , Moving Screen Use Guidelines: Nine Reasons Why Screen Use Guidelines Should Be Separated From Public Health 24‐Hour Movement Guidelines in Australia and Internationally. Digital Child Working Paper 2023–01 (ARC Centre of Excellence for the Digital Child, 2023).

[15] P. Nikken and M. Schols , “How and Why Parents Guide the Media Use of Young Children,” Journal of Child and Family Studies 24, no. 11 (2015): 3423–3435. 26472932 10.1007/s10826-015-0144-4 PMC4598347

[16] C. S. Wu , C. Fowler , W. Y. Lam , H. T. Wong , C. H. Wong , and A. Yuen Loke , “Parenting Approaches and Digital Technology Use of Preschool Age Children in a Chinese Community,” Italian Journal of Pediatrics 40 (2014): 44. 24887105 10.1186/1824-7288-40-44 PMC4046626

[17] E. Rivera , K. D. Hesketh , L. Orellana , et al., “Prevalence of Toddlers Meeting 24‐Hour Movement Guidelines and Associations With Parental Perceptions and Practices,” Journal of Science and Medicine in Sport 27, no. 4 (2024): 250–256. 38216403 10.1016/j.jsams.2023.12.008

[18] S. Houghton , S. C. Hunter , M. Rosenberg , et al., “Virtually Impossible: Limiting Australian Children and Adolescents Daily Screen Based Media Use,” BMC Public Health 15 (2015): 5. 25613954 10.1186/1471-2458-15-5 PMC4324783

[19] L. Tooth , K. Moss , R. Hockey , and G. D. Mishra , “Adherence to Screen Time Recommendations for Australian Children Aged 0–12 Years,” Medical Journal of Australia 211, no. 4 (2019): 181–182. 31334841 10.5694/mja2.50286

[20] B. A. Bruijns , M. Bourke , K. Saravanamuttoo , and P. Tucker , “Physical Activity, Sedentary Behaviour, and Sleep Knowledge and Self‐Efficacy Among Parents of Young Children in Canada,” Journal of Activity, Sedentary and Sleep Behaviors 3, no. 1 (2024): 12. 38764474 10.1186/s44167-024-00051-x PMC11101324

[21] M. Howard and S. A. Akhund , “Parents' Knowledge, Perceptions and Support Around Appropriate Physical Activity, Screen Time and Sleep Time Levels for Children,” International Journal of Child Care and Education Policy 18, no. 1 (2024): 2.

[22] S. Kerai , A. Almas , M. Guhn , B. Forer , and E. Oberle , “Screen Time and Developmental Health: Results From an Early Childhood Study in Canada,” BMC Public Health 22, no. 1 (2022): 310. 35168575 10.1186/s12889-022-12701-3 PMC8845249

[23] Y. Chassiakos , J. Radesky , D. Christakis , et al., “Children and Adolescents and Digital Media,” Pediatrics 138, no. 5 (2016): e20162593. 27940795 10.1542/peds.2016-2593

[24] A. Morawska , A. E. Mitchell , and L. R. Tooth , “Managing Screen Use in the Under‐Fives: Recommendations for Parenting Intervention Development,” Clinical Child and Family Psychology Review 26, no. 4 (2023): 943–956. 37171529 10.1007/s10567-023-00435-6 PMC10640456

[25] G. O. Boateng , T. B. Neilands , E. A. Frongillo , H. R. Melgar‐Quiñonez , and S. L. Young , “Best Practices for Developing and Validating Scales for Health, Social, and Behavioral Research: A Primer,” Frontiers in Public Health 6 (2018): 149. 29942800 10.3389/fpubh.2018.00149 PMC6004510

[26] Australian Bureau Statistics , Census of Population and Housing: Socio‐Economic Indexes for Areas (SEIFA) (Commonwealth of Australia, 2016).

[27] J. Cohen , Statistical Power Analysis for the Behavioral Sciences, 2nd ed. (Routledge, 1988).

[28] T. Deave , D. Johnson , and J. Ingram , “Transition to Parenthood: The Needs of Parents in Pregnancy and Early Parenthood,” BMC Pregnancy and Childbirth 8 (2008): 30. 18664251 10.1186/1471-2393-8-30 PMC2519055

[29] D. Hendry , L. Straker , B. Bourne , et al., “Parental Practices and Perspectives on Health and Digital Technology Use Information Seeking for Children Aged 0–36 Months,” Health Promotion Journal of Australia 35, no. 4 (2024): 1174–1183. 38382122 10.1002/hpja.849

[30] Healthdirect Australia , Routine Health Checks for Babies and Children (Australian Government Department of Health and Aged Care, 2023), https://www.pregnancybirthbaby.org.au/health‐checks‐for‐babies‐and‐children.

[31] Queensland Government , Crisafulli Government Delivers Free Health Checks for Queensland Kids (Queensland Government, 2024), https://statements.qld.gov.au/statements/102786.

[32] T. Alsop , E. Lehman , S. Brauer , et al., “What Should All Health Professionals Know About Movement Behaviour Change? An International Delphi‐Based Consensus Statement,” British Journal of Sports Medicine 57 (2023): 1419–1427. 37793699 10.1136/bjsports-2023-106870

[33] C. Leaper , “Parents' Socialization of Gender in Children,” in Encyclopedia on Early Childhood Development, ed. C. L. Martin , R. E. Tremblay , M. Boivin , and R. D. V. Peters (Centre of Excellence for Early Childhood Development, 2013), 1–5, http://www.child‐encyclopedia.com/documents/LeaperANGxp1.pdf.

[34] S. Madigan , B. A. McArthur , C. Anhorn , R. Eirich , and D. A. Christakis , “Associations Between Screen Use and Child Language Skills: A Systematic Review and Meta‐Analysis,” JAMA Pediatrics 174, no. 7 (2020): 665–675. 32202633 10.1001/jamapediatrics.2020.0327 PMC7091394

[35] L. Goodall , “Enhancing Parental Engagement With Children's Learning Through Accessible Communication of Guidance,” Education Review 33, no. 2 (2021): 133–150.

