# Supplementary material for: Parental Knowledge, Attitudes, and Perceived Usefulness of Australian Screen Time Guidelines for Toddlers and Preschoolers
Source: Health Promot J Austr. 2026 Mar 30;37(2):e70178. doi: 10.1002/hpja.70178 (PMC13036367; doi:10.1002/hpja.70178)
Supplement: Supplementary file 1 — Table S1: presents survey items and responses assessing parental knowledge of Australian screen time guidelines. [file HPJA-37-0-s001.docx]

**Supplementary Table 1. Parental knowledge of Australian screen time guidelines: response options and answers**

| **Question:** What are the current Australian screen time guidelines for your youngest child aged 1 year or above? | |
| --- | --- |
| **Response options - Toddlers** | **N (%)** |
| For those younger than 2 years, sedentary screen time is not recommended. For those aged 2 years, sedentary screen time should be no more than 1 hour per day; less is better. (Correct response) | 106 (86.9) |
| For those younger than 2 years, no more than 1 hour of sedentary screen time per day is recommended. For those aged 2 years, sedentary screen time should be no more than 2 hours per day. | 5 (4.1) |
| For those younger than 2 years, no more than 2 hours of sedentary screen time per day is recommended. For those aged 2 years, sedentary screen time should be no more than 3 hours per day. | 0 (0.0) |
| I don’t know | 11 (9.0) |
| **Response options - Preschoolers** | **N (%)** |
| Sedentary screen time should be no more than 1 hour per day; less is better. (Correct response) | 64 (59.8) |
| Sedentary screen time should be no more than 2 hours per day. | 18 (16.8) |
| Sedentary screen time should be no more than 3 hours per day. | 2 (1.9) |
| I don’t know | 23 (21.5) |
